# Deletion of *cyp125* Confers Increased Sensitivity to Azoles in *Mycobacterium tuberculosis*

**DOI:** 10.1371/journal.pone.0133129

**Published:** 2015-07-21

**Authors:** Paul Carroll, Tanya Parish

**Affiliations:** 1 Queen Mary University of London, Barts & The London School of Medicine and Dentistry, Centre for Infectious Disease, London, United Kingdom; 2 TB Discovery Research, Infectious Disease Research Institute, Seattle, Washington, United States of America; Institut de Pharmacologie et de Biologie Structurale, FRANCE

## Abstract

*Mycobacterium tuberculosis* is able to utilize cholesterol as a carbon source, and this ability is linked to its virulence in macrophages and in the mouse model of infection. The *M*. *tuberculosis* cytochrome P450 Cyp125 plays a key role in cholesterol metabolism being involved in the first steps of its degradation. Cyp125 is a cholesterol hydroxylase which is essential for cholesterol catabolism in *M*. *bovis* BCG and some strains of *M*. *tuberculosis*. We generated an unmarked, in-frame deletion of Cyp125 in *M*. *tuberculosis* H37Rv. The deletion strain was able to grow as well as wild-type in medium containing glucose as the carbon source. The Cyp125 deletion strain was more sensitive to growth inhibition by clotrimazole consistent with the ability of Cyp125 to bind azoles with high affinity. The deletion strain showed no difference in sensitivity to nitric oxide or hydrogen peroxide and was not attenuated for growth inside THP-1 human macrophage-like cells. These data suggest that the attenuation of virulence seen in operon deletion strains is not linked to the lack of Cyp125 alone.

## Introduction

*Mycobacterium tuberculosis*, the causative agent of tuberculosis, is responsible for the death of over 1 million people and 9 million new cases per year [1]. The current treatment of tuberculosis is time consuming and with the current treatment procedures there is an increase in the number of both multidrug resistant (MDR-TB) and extensively drug resistant (XDR-TB) *M*. *tuberculosis* strains [1]. This indicates a need for an increased understanding of the biology and pathogenic mechanisms of the bacterium in order to develop new therapeutic strategies.

Unusually, the *M*. *tuberculosis* genome encodes 20 cytochrome P450 enzymes (Cyps) [2,3]. Among the mycobacteria, the closely related species *M*. *bovis* has 18, and the non-pathogenic species *M*. *smegmatis* has 39 [4]. In relation to other bacteria this is a relatively high number, since very few prokaryotes encode any Cyp homologues; for example, *Escherichia coli* has none [4,5]. In contrast, *M*. *leprae* has only one, which may represent the minimal requirement for a mycobacterium, since it has undergone substantial reductive evolution of its genome [6].

P450s are of interest as drug targets, for example the polycyclic azoles are P450 inhibitors which have been used topically to combat fungal pathogens. Azoles inhibit Cyp51, an essential lanosterol 14α–demethylase, involved in the synthesis of ergosterol—an integral component of the cell membrane [7]. Azoles also have anti-mycobacterial properties, with activity against *M*. *tuberculosis* both *in vitro* and *in vivo* in the mouse model of infection [8–12]. Unfortunately, azoles have hepatotoxic and teratogenic properties, which precludes their use in long term treatment for tuberculosis [13]. The main mechanism of resistance to azoles is increased drug efflux, mediated in *M*. *tuberculosis* by the MmpS5-MmpL5 system [14–16].

Cyp125 plays a role in cholesterol metabolism in *M*. *tuberculosis* [17,18]. Cholesterol degradation is required for both the spread and persistence of the bacteria *in vivo* [19–21]. In addition, nitric oxide has been shown to bind to Cyp125, and other P450 enzymes, and a role in removing dangerous free radicals has been suggested [22].

Cyp125 catalyses the C27 hydroxylation of both cholesterol and choles-4-ten-3-one, although its deletion results in accumulation of the latter metabolite which is presumed to be toxic to the cells [23–25]. Cyp125 forms part of the *igr* locus [24,25] and is found in a region of the genome with other genes encoding enzymes also involved in cholesterol degradation [26]. In *M*. *bovis* BCG, *Rhodococcus jostii* RHA1 and *M*. *tuberculosis* CDC1551, Cyp125 is essential for growth on cholesterol [18,23,27]. In contrast in *M*. *tuberculosis* H37Rv, a Cyp125 deletion mutation was able to grow on cholesterol as the only carbon source [18] and this was linked to the ability of Cyp142 to provide similar C27 hydroxylation capability [28].

## Materials and Methods

### Bacterial Strains, Growth Media and Antibiotics

*M*. *tuberculosis* H37Rv (ATCC25618) was grown in Middlebrook 7H9 medium plus 10% v/v OADC supplement (Becton Dickinson) and 0.05% w/v Tween 80 or on Middlebrook 7H10 agar plus 10% v/v OADC. Hygromycin was added at 100 μg/ml, kanamycin at 20 μg/ml, gentamicin at 10 μg/ml, X-gal at 50 μg/ml.

### Generation of a *M*. *tuberculosis* CYP125 Deletion Strain

A deletion delivery vector was generated by amplifying the upstream and downstream regions of Rv3545c, encoding Cyp125, using primer pairs F1 5’ AAG CTT ACG AAG ATC TGC TGC TCG AT 3’ and R1 5’ GGA TCC CAC TGG CAG GTC GAC TAC ACC 3’, and F2 5’ GGA TCC CTC CAC TGA CTG GTG ATT CCA3 ‘ and R2 5’ GCG GCC GCT CGT TGA TCT CGA CGA TGT 3’ and cloned into p2NIL [29] as a HindIII-NotI fragment to generate an unmarked in-frame deletion. Restriction sites used for cloning are underlined. The gene cassette from pGOAL19 [29] was cloned in as a PacI fragment to generate the final delivery vector pTACK125. The deletion delivery vector pTACK125 was electroporated into *M*. *tuberculosis* and single cross-overs (SCOs) were isolated. Double cross-overs (DCOs) were isolated from the SCO strain as previously described [29]. DCOs were screened for the presence of the wild-type or deletion allele using primers CYP125D1 5’ CGT CTG AAC CAT TCG ATG TG 3’ and CYP125D2 5’ TTC AAC GAT GAC CGG GTA AC, which amplify a product of 2.0 kb from the wild-type and 0.7 kb from the deletion. The deletion strain was confirmed by Southern blotting (S1 Fig). A complementing vector (pCOLE125) was constructed by amplifying the Rv3545c gene using primers Cyp125D1 and Cyp125D2, cloning into pSC-A (Stratagene), and adding the integrating cassette (Gm, attP, L5 int) from pUC-Gm-INT [30] as a HindIII fragment to generate an integrating vector with gentamicin resistance.

### Growth Curves and Cell-Free Extracts

Liquid cultures were diluted to give a starting OD_580_ of 0.01 in 3 mL of medium. Each tube contained a 12 mm magnetic stirrer and was incubated at 37°C on a Wheaton Biostir. OD_580_ readings were taken periodically.

### Macrophage Infection Assays

Macrophages were prepared and infected with *M*. *tuberculosis* as described previously [31]. Human THP-1 monocyte macrophages [32] were differentiated with 5 ng/mL PMA for 3 days and infected at an MOI of 1:1. To activate the monocytes, IFN-γ was added at 100 unit ml^-1^ and incubated for 24 hours. Bacteria were harvested periodically post infection and the CFUs determined.

## Results

We were interested in the role of P450s in the metabolism of *M*. *tuberculosis*. Cyp125 is of interest as it may play a role in virulence. We constructed an in-frame, unmarked deletion strain in the *M*. *tuberculosis* H37Rv (London Pride) background [33] using a two-step homologous recombination method. The strain (Tame 127) was confirmed by Southern blotting to possess the expected genotype (S1 Fig).

### *Cyp125* Deletion Does Not Impair Growth in Liquid Medium with Glucose as the Carbon Source

**Fig 1:**
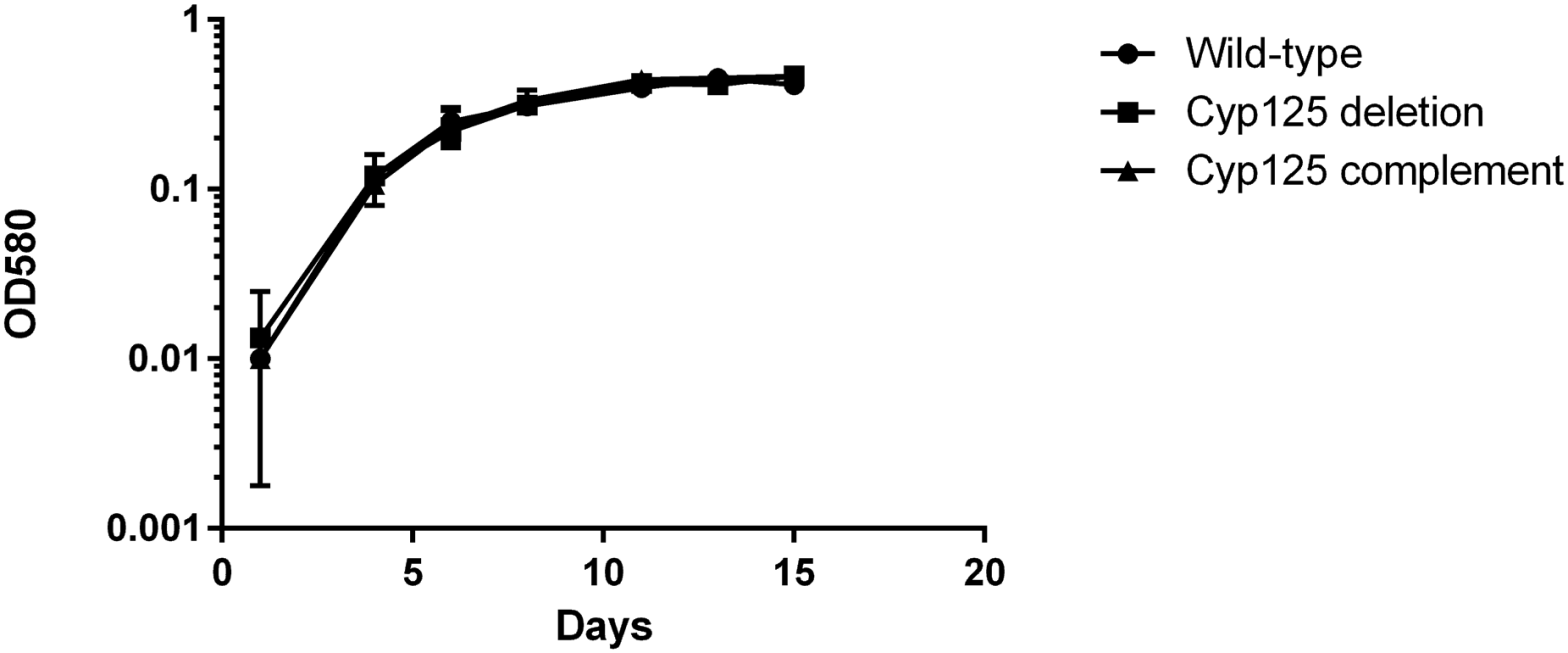
Growth of the *M*. *tuberculosis cyp125*Δ strain in liquid medium with carbon as the glucose source. Strains were inoculated into liquid medium containing glucose as the sole carbon source. Square—wild-type; circle–*cyp125*Δ strain; triangle—complemented strains. Data are the mean and standard deviation of three independent cultures.

We first looked at the growth of the deletion strain in liquid medium as compared to the wild-type strain. No difference was seen in the growth rate between strains (Fig 1). In addition, since the deletion strain was obtained on 7H10 medium with OADC supplement i.e. glucose as the carbon source, it confirmed that the knockout strain was able to utilize this carbon source. Since we did not expect Cyp125 to be involved in this aspect of metabolism, this was not surprising.

### *Cyp125* Deletion Has No Effect on Survival in Nitric Oxide

**Fig 2:**
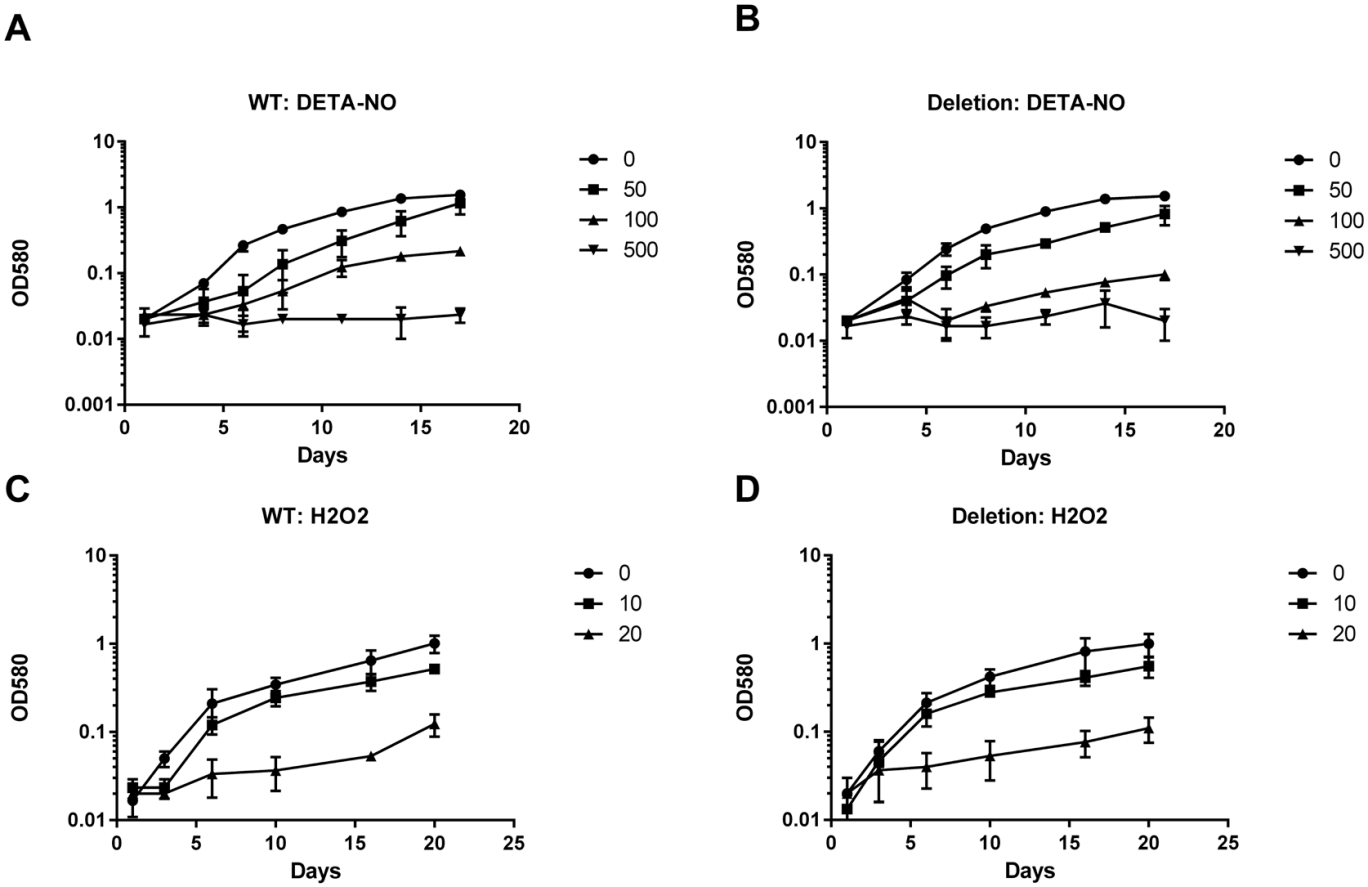
Growth of *M*. *tuberculosis* in the presence of nitric oxide or hydrogen peroxide. Strains were inoculated into liquid medium containing DETA-NO to generate NO (A and B), or hydrogen peroxide (C and D). A and C—wild type; B and D–*cyp125Δ* strain. DETA-NO concentrations were: diamond– 0; square 50 μM; triangle 100 μM; cross 200 μM. Hydrogen peroxide concentrations were: diamond– 0; square 10 μM; triangle– 20 μM. Data are the mean and standard deviation of three independent cultures.

*M*. *tuberculosis* P450s have different sensitivity to nitric oxide-mediated inhibition [22]. Cyp125 reversibly binds with nitric oxide, the interaction is labile such that the reduced ferrous-NO complex is converted back to the ferric state after exposure to oxygen [22]. This suggests that Cyp125 would be more resistant to NO inhibition. We wanted to test if deletion of Cyp125 leads to increased susceptibility to NO. We tested the effect of NO, as generated by the donor DETA-NO, on growth kinetics. As expected, increasing concentrations of NO led to a decrease in growth rate, until complete inhibition was reached at the highest concentration. We saw no difference in the growth kinetics of the mutant as compared to the wild-type strain (Fig 2). We also tested the effect of hydrogen peroxide on growth; again we saw marked inhibition of growth which was dose dependent, but there was no increased susceptibility in the Cyp125Δ strain. These data indicate that Cyp125 plays no role in combating these stresses in this model.

### The *cyp125Δ* Strain Is Fully Virulent in the Macrophage Model of Infection

**Fig 3:**
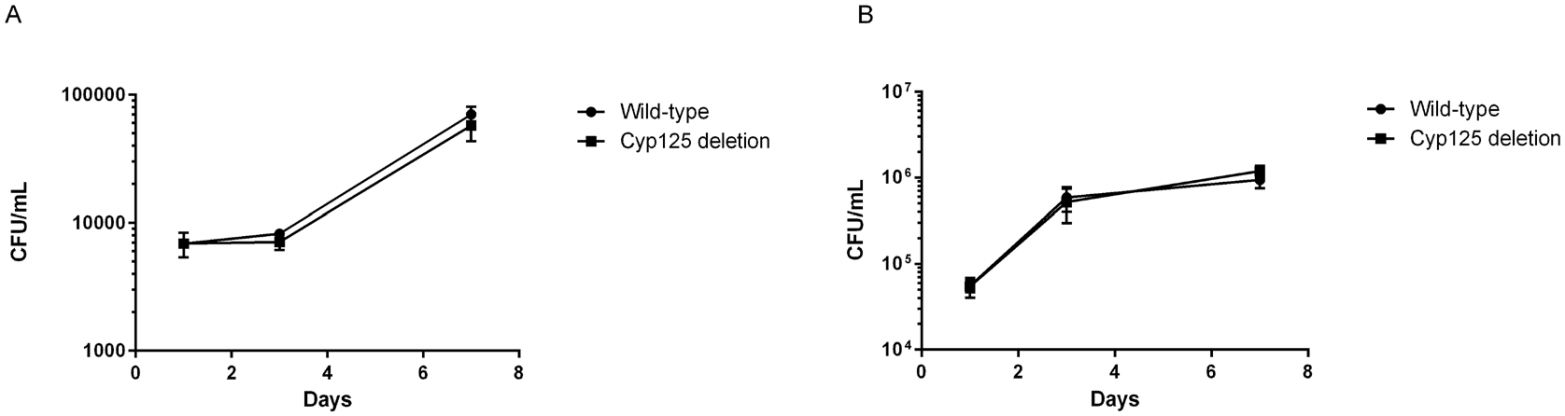
Virulence of *M*. *tuberculosis* Cyp125Δ during intracellular infection. THP-1 cells were infected with *M*. *tuberculosis* strains and bacterial survival monitored over 7 days by CFUs. (A) Resting macrophages. (B) Activated macrophages. Data are the mean and standard deviation from three independent infections.

Cyp125 is induced in the macrophage model of infection, suggesting that it plays an important role in virulence, presumably related to its role in cholesterol degradation [19,34]. The operon containing Cyp125 is essential for virulence in resting macrophages, although it was not determined if Cyp125 itself was required [24,25]. We wanted to determine if Cyp125 was required for replication in macrophages; we tested the mutant for growth/survival in resting and activated human monocytes (Fig 3). The deletion strain grew to the same extent as the wild-type strain in both conditions. Activation of macrophages resulted in a small restriction of growth of bacteria over 7 days for both strains. These data indicate that Cyp125 is not required for intracellular multiplication.

### The *cyp125Δ* Strain Has Increased Sensitivity to Azoles

**Fig 4:**
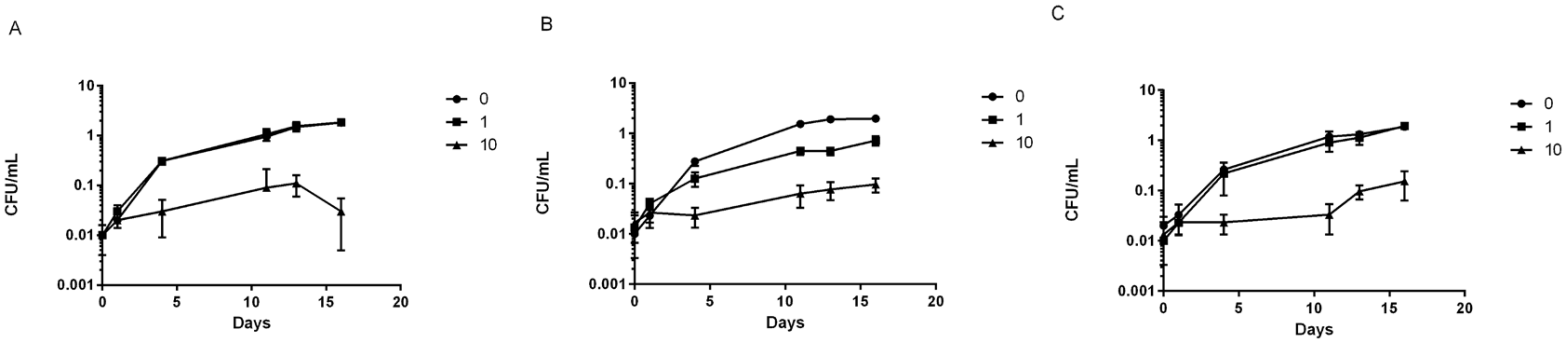
Sensitivity of *M*. *tuberculosis cyp125*Δ strain to azole inhibition of growth. Strains were inoculated into liquid medium containing clotrimazole at 1 and 10 μg/ml and growth compared to untreated (no clotrimazole = 0). A—wild-type strain; B–*cyp125* deletion strains; C—complemented strain, Data are the mean and standard deviation of three independent cultures.

We had previously noted that a Cyp144 deletion strain was more sensitive to clotrimazole and econazole [35]. Since azoles do bind to Cyp125, we predicted that the deletion strain might also be more sensitive to growth inhibition. Once again we saw that the deletion strain was more sensitive to inhibition by clotrimazole (Fig 4). The complemented strain had the same sensitivity as the wild-type, confirming this was due to *cyp125* deletion and not a polar effect. Since azoles are subject to efflux, and resistance is mediated by efflux, we also looked at the effect of efflux inhibitors (CCCP, reserpine, and verapamil) in combination with clotrimazole. Addition of any of the efflux inhibitors had no effect on clotrimazole sensitivity in any of the strains (data not shown), suggesting, that at least for this azole, efflux does not influence the intracellular accumulation sufficiently to affect activity. No differences were seen between the deletion strain and the wild type (data not shown).

## Discussion

We were able to generate an in-frame, unmarked deletion of Cyp125 in *M*. *tuberculosis* H37Rv; this confirms previous work that the gene is not essential under standard laboratory growth conditions [18,23–25]. Deletion of *cyp125* had no effect on growth in glucose or stress response. The *cyp125* deletion strain was not attenuated in the macrophage model. Previous work suggests that deletion of the *igr* locus (rv3540c-Rv3545c) resulted in attenuation [24,25], but we show this is not linked to *cyp125* deletion, but presumably to the other genes in the operon, or a combination of them.

We found that deletion of *cyp125* also conferred increased sensitivity to clotrimazole, which was not dependent on efflux. Cyp125 has a high affinity for azoles, and so may play a role in azole resistance by titrating out the compound in competition with the real cellular target. In support of this hypothesis, we found that deletion of Cyp144 similarly led to increased sensitivity [35], suggesting a non-specific mechanism. No target for azole action in *M*. *tuberculosis* has yet been found. Our data support the idea that although P450s play a role in binding azoles (and may be secondary targets), they are not the major target of action of this class.

## Supporting Information

S1 Fig Southern analysis of double cross-overs. Several DCO strains were selected for analysis. Genomic DNA was isolated, digested with BamHI, separated on an 0.8% w/v agarose gel, transferred to a blotting membrane and probed with cyp125 (PCR product generated using primers D1 and D2). The wild-type genomic restriction map and Southern probe is shown. Expected sizes for the wild-type were 2.2, 1.8 and 0.4 kb (double band). Expected sizes for the deletion were 2.0 and 1.6 kb. Lanes 3,6,7,0 had deletion alleles. Lanes 2,4,5,8 had wild-type alleles. Lane 1–1 kb markers. The strain from Lane 3 was selected for studies.
(PPTX)
