# Supplementary material for: Deletion of cyp125 Confers Increased Sensitivity to Azoles in Mycobacterium tuberculosis
Source: PLoS One. 2015 Jul 21;10(7):e0133129. doi: 10.1371/journal.pone.0133129 (PMC4510303; doi:10.1371/journal.pone.0133129)
Supplement: S1 Fig — Several DCO strains were selected for analysis. Genomic DNA was isolated, digested with BamHI, separated on an 0.8% w/v agarose gel, transferred to a blotting membrane and probed with cyp125 (PCR product generated using primers D1 and D2). The wild-type genomic restriction map and Southern probe is shown. Expected sizes for the wild-type were 2.2, 1.8 and 0.4 kb (double band). Expected sizes for the deletion were 2.0 and 1.6 kb. Lanes 3,6,7,0 had deletion alleles. Lanes 2,4,5,8 had wild-type alleles. Lane 1–1 kb markers. The strain from Lane 3 was selected for studies. (PPTX) [file pone.0133129.s001.pptx]

## Slide 1
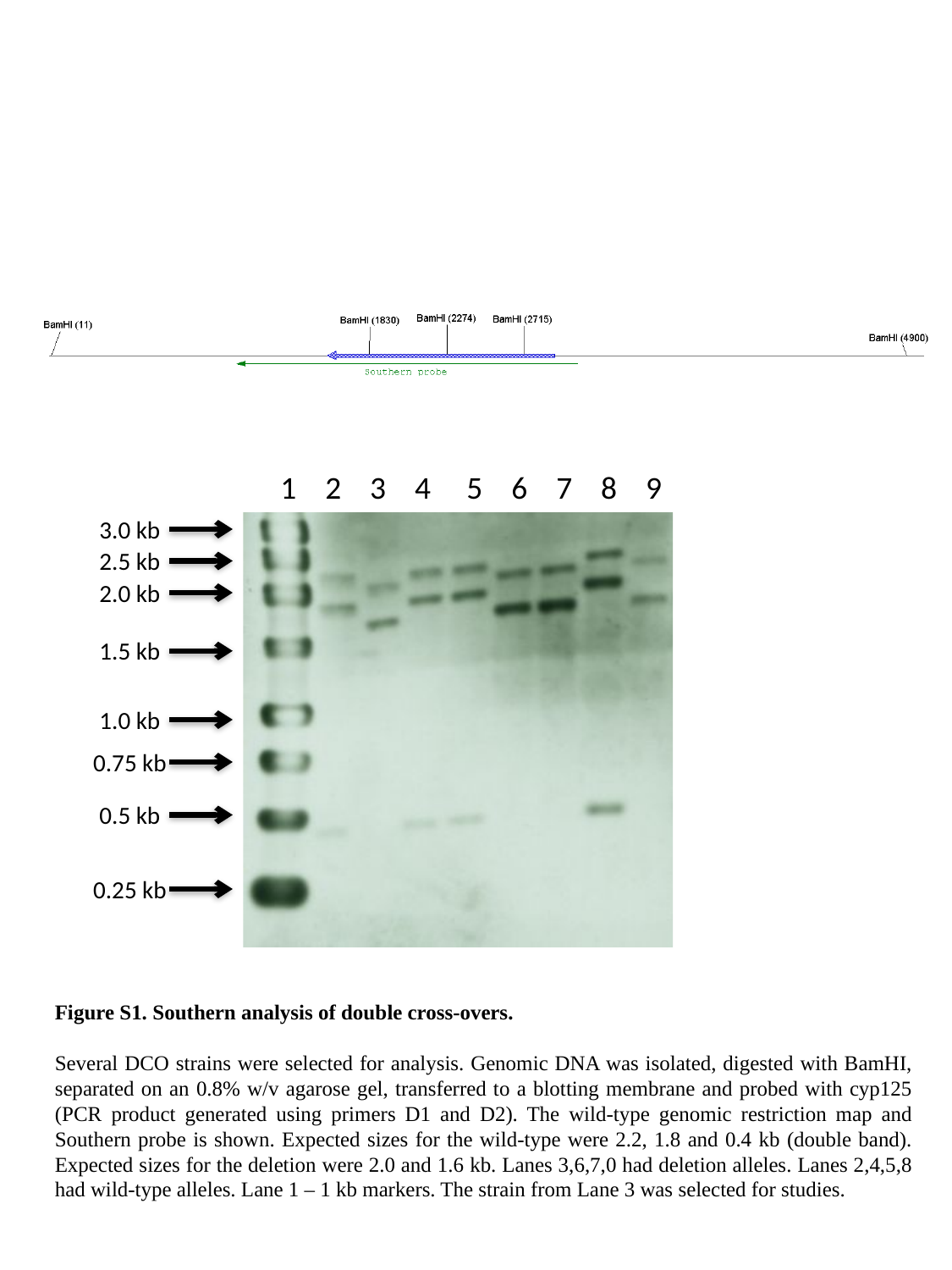

1 2 3 4 5 6 7 8 9
3.0 kb
2.5 kb
2.0 kb
1.5 kb
1.0 kb
0.75 kb
0.5 kb
0.25 kb
Figure S1. Southern analysis of double cross-overs.
Several DCO strains were selected for analysis. Genomic DNA was isolated, digested with BamHI, separated on an 0.8% w/v agarose gel, transferred to a blotting membrane and probed with cyp125 (PCR product generated using primers D1 and D2). The wild-type genomic restriction map and Southern probe is shown. Expected sizes for the wild-type were 2.2, 1.8 and 0.4 kb (double band). Expected sizes for the deletion were 2.0 and 1.6 kb. Lanes 3,6,7,0 had deletion alleles. Lanes 2,4,5,8 had wild-type alleles. Lane 1 – 1 kb markers. The strain from Lane 3 was selected for studies.
